# Patients with atrial fibrillation and permanent pacemaker: Temporal changes in patient characteristics and pharmacotherapy

**DOI:** 10.1371/journal.pone.0195175

**Published:** 2018-03-28

**Authors:** Frederik Dalgaard, Martin H. Ruwald, Tommi Bo Lindhardt, Gunnar H. Gislason, Christian Torp-Pedersen, Jannik L. Pallisgaard

**Affiliations:** 1 Department of Cardiology, Copenhagen University Hospital, Herlev and Gentofte Hospital, Hellerup, Denmark; 2 Danish Heart Foundation, Copenhagen, Denmark; 3 The National Institute of Public Health, University of Southern Denmark, Odense, Denmark; 4 Department of Health Science and Technology, Aalborg University Hospital, Aalborg, Denmark; Indiana University, UNITED STATES

## Abstract

**Background:**

The management of patients with non-valvular atrial fibrillation (NVAF) with rate-lowering or anti-arrhythmic drugs has markedly changed over the last decade, but it is unknown how these changes have affected patients with NVAF with a permanent pacemaker (PPM).

**Methods:**

Through Danish nationwide registries, patients with NVAF and a PPM were identified from 2001 to 2012. Changes in concomitant pharmacotherapy and comorbidities were tested using the Cochran–Armitage trend test and linear regression. Patients with NVAF were identified to calculate the proportional amount of PPM implants.

**Results:**

A total of 12,231 NVAF patients with a PPM were included in the study, 55.6% of which were men. Median age was 78 years (interquartile range 70–84). From 2001 to 2012, the number of NVAF patients with a PPM increased from 850 to 1344, while the number of NVAF patients increased from 67,478 to 127,261. Thus, the proportional amount of NVAF patients with a PPM decreased from 1.3% to 1.1% (p = 0.015). Overall 45.9% had atrial fibrillation (AF) duration less than one year and the proportion declined from 55.5% to 42.4% (p <0.001). Diabetes mellitus increased from 7.2% to 16.8% (p <0.001). Heart failure (HF) decreased from 36.7% to 29.3% (p = 0.010) and ischemic heart disease (IHD) decreased from 32.4% to 26.1% (p <0.001). Beta-blocker use increased from 38.1% to 58.0% (p <0.001), while digoxin and anti-arrhythmic drug use decreased over time.

**Conclusion:**

From 2001 to 2012, the absolute number of NVAF patients with a PPM increased while the proportional amount decreased. The number of NVAF patients receiving a PPM within one year of AF diagnosis decreased. The prevalence of DM increased, while the prevalence of HF and IHD was high but decreasing. The use of beta-blockers increased markedly, while use of digoxin and anti-arrhythmic drugs decreased over time.

## Introduction

Treatment strategies of non-valvular atrial fibrillation (NVAF) have changed over the last decade, potentially resulting in changes to the need for permanent pacemaker (PPM) implantation [1–4]. Various conditions warrant a PPM in patients with NVAF to prevent symptoms and syncope, death, and to improve quality of life, the most common being symptomatic bradycardia induced by either sick sinus node syndrome or iatrogenic due to the prescription of rate-lowering or anti-arrhythmic drugs [5–8]. Atrial fibrillation (AF) is the most common arrhythmia and during the last 10 years emerging evidence has estimated the prevalence in the adult population to be around 2.3–3.4% with an increasing incidence [9,10]. Immigrant studies and prevalence studies suggests that AF is more common in the Nordic countries [11,12]. Furthermore, the incidence of AF in Denmark has increased threefold during the last 30 years [13]. Despite of these data, it is unknown to which extent the increase in AF incidence have affected the use of PPM implants in Denmark. Secondly, it is unknown how the patients with NVAF and PPM are treated at the time of device implantation. Furthermore, it has not been described whether the temporal changes in comorbidity burden and use of pharmacotherapy during the last decade among these patients reflect the general trends among NVAF patients. The aim of this study was to investigate the temporal trend of the proportional amount of PPM implants in NVAF patients and to investigate the temporal changes in patient demographics, concomitant use of pharmacotherapy, cardiovascular surgical procedures, and comorbidities in NVAF patients with a PPM from 2001 to 2012.

## Methods

All Danish residents are, at birth or migration, provided with a unique civil registration number enabling crosslinking between nationwide registers. In this retrospective nationwide cohort study, The Civil Registration System, The Danish National Patient Register (DNPR), The Integrated Database for Labor Market Research Database, and The Danish Drug Statistical Registry were used to identify patient demographics, medical procedures, comorbidities, annually income, educational level, and concomitant pharmacotherapy. The Civil Registration System holds data on age, sex, and vital status. DNPR holds information on every hospital admission in Denmark since 1977. Each hospitalization is registered at date of admission and contains information on the date of discharge combined with one primary diagnosis and, if applicable, one or more secondary diagnoses defined by the International Classification of Diseases; the 8^th^ (ICD-8) or the10^th^ revision (ICD-10) since 1994. DNPR also holds information on medical procedures including PPM implantations performed in Denmark. Procedures have been registered since 1996 and coded by the Nordic Classification of Surgical Procedures (NCSP). The Integrated Database for Labor Market Research Database contains information on annual household income since 1980 and information on the highest individually achieved educational level. The Danish Drug Statistical Registry holds information on all drug prescriptions redeemed since 1995. Each drug is classified by the international Anatomical Therapeutic Chemical (ATC) classification [14].

### Study cohort

Patients with NVAF were included in the study on the date of PPM implantation from 1^st^ January, 2001 to 31^st^ December 2012. Single chamber atrial PPM, single chamber ventricular PPM, dual chamber PPMs, cardiac resynchronization therapy (CRT) with pacemakers (CRT-P), and CRT with implantable cardioverter-defibrillator (CRT-D) were identified using NCSP procedure codes. The NCSP codes have previously been validated [15]. NVAF was defined as the diagnosis of AF by ICD-10 or ICD-8 codes I48, 42794, 42795 with absence of rheumatic valve disease and mechanical valve replacement as previously done [16]. Data on AF type (paroxysmal, persistent or permanent) was unavailable. The patients were divided into groups by year of PPM implantation date between 2001 and 2012. Annually income was defined as household income after taxation and interest and for the value of the Danish currency in 2009. Income was estimated as an average of the year of PPM and up to 5 years prior. For categorical analysis, income was grouped into four groups; < 1^st^. quartile, 1^st^-2^nd^ quartile, 2^nd^-3^rd^ quartile, and ≥3^rd^ quartile. Income groups were also divided at age ≥65 years and <65 years, to consider for those in the working age. Three educational levels were grouped as follows: basic and high school, vocational education, and higher education (university degree). AF duration was identified as the time between date of first AF diagnosis and date of PPM implantation. NCSP codes were used to identify coronary artery bypass grafting, percutaneous coronary intervention, radiofrequency catheter ablations for AF, and cardioversion. ICD-10 codes were used to identify heart failure (HF), ischemic heart disease (IHD), ischemic stroke, chronic obstructive pulmonary disease, sick sinus node syndrome, atrio-ventricular (AV) block of all types, and unspecified bradycardia.

Procedures and comorbidities were identified for each patient within 5 years prior to the date of inclusion (baseline date). ATC codes were used to identify prescription drugs claimed within 180 days prior to date of inclusion for beta-blockers, digoxin, class 4 calcium channel blockers (diltiazem and verapamil), amiodarone, class 1C (flecainide and propafenone), non-loop diuretics, loop-diuretics, calcium channel blockers, renin-angiotensin system inhibitors, and oral anticoagulants (antagonists and non-vitamin-K antagonists). Anti-diabetic drugs were used as a proxy for diabetes mellitus (DM). All ICD-10 codes, ICD-8 codes, NCSP codes, and ATC codes used are available in S2 Table. Three subgroup analyses of patients with IHD, DM, and HF were performed. Besides the study cohort, the total number of alive patients with NVAF for each year from 2001 to 2012 was identified. These were defined as patients with NVAF and the AF diagnosis on a date before or within 2001 adding new incident patients with NVAF each subsequent year up to and including 2012.

### Statistical analysis

Categorical data were presented as counts with percentages and statistical differences were tested using a Chi-squared test. Continuous variables were presented as medians with interquartile range (IQR) and statistical differences were tested using a Kruskal-Wallis test. The proportional amount of NVAF patients with a PPM was calculated by the number of NVAF patients with a PPM out of the total number of patients with NVAF for each year. Trends in categorical variables were tested for using a Cochran–Armitage test and for continuous variables, trends were tested for using linear regression analysis. A two-sided p-value <0.05 was considered statistically significant. Data management and statistical analyses were conducted using R statistics (R Core Team (2016). R: A language and environment for statistical computing. R Foundation for Statistical Computing, Vienna, Austria. URL http://www.R-project.org/).

### Ethics

In Denmark, retrospective register studies do not require approval from ethics committees. The Danish Data Protection Agency approved this study (ref. no.: 2007-58-0015/GEH-2014-016 I-Suite no.: 02734). Data were made available to us in an anonymized format such that specific individuals could not be identified.

## Results

### Study population

**Fig 1:**
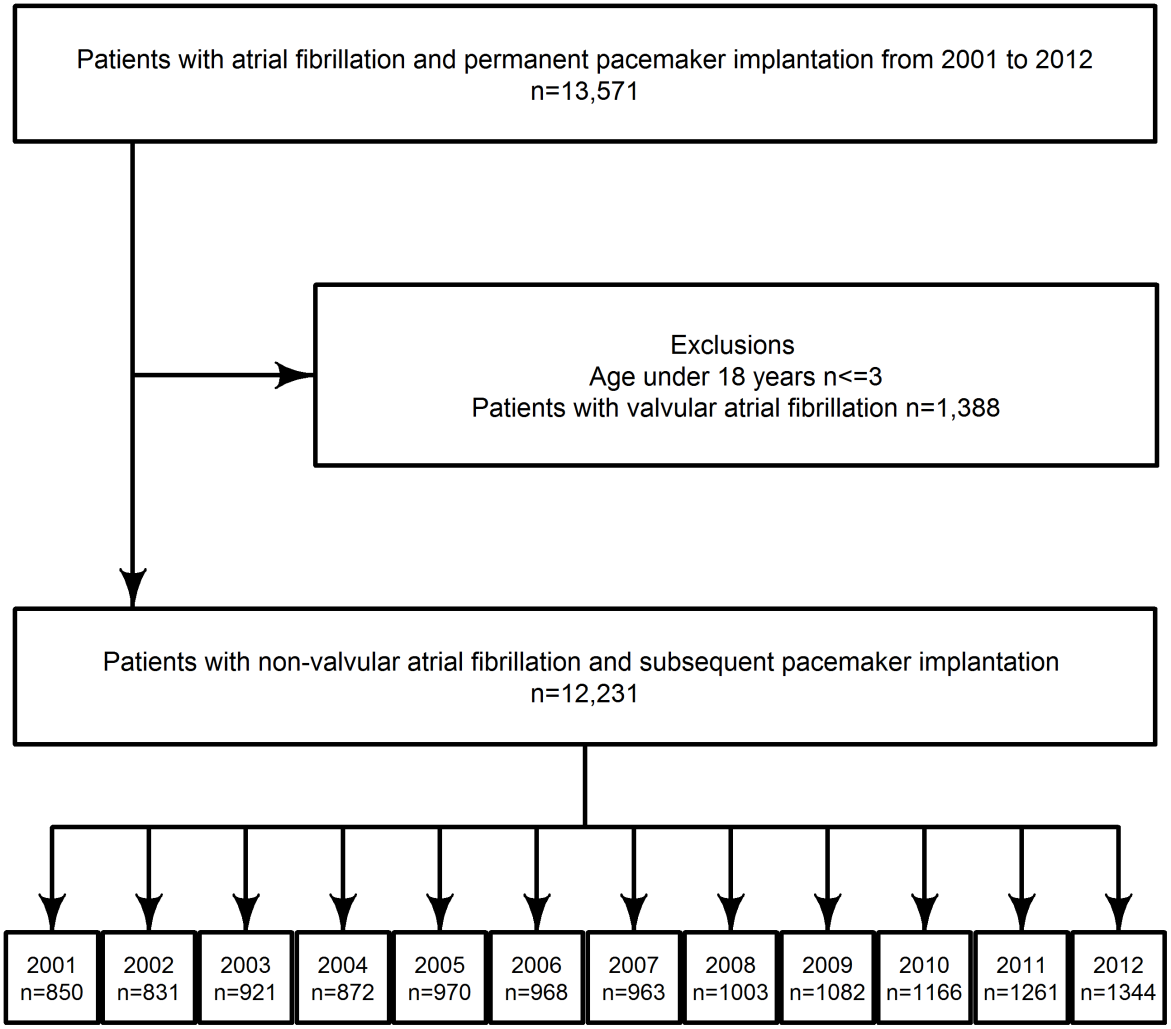
Flowchart. Flowchart inclusion of the study cohort. Stratified by year of pacemaker implantation date.

A total of 12,231 patients with NVAF and PPMs were included between 2001 and 2012 (Fig 1). The overall median age (IQR) was 78 (70–84) with most patients being men (55.6%, n = 6,805). The median (IQR) duration of AF prior to PPM implant was 1.39 years (0.07–5.09) and a total of 45.9% (n = 5,615) of the patients received a PPM within the first year of their AF diagnosis. The PPM type mostly used was the dual chamber pacemaker accounting for 39.4% (n = 4,823). Overall prevalence of HF, IHD, and DM was 33.6% (n = 4,104), 29.6% (n = 3,621) and 13.9% (n = 1,704), respectively. The most common bradyarrhythmia diagnosis was sick sinus node syndrome with a prevalence of 43.7% (n = 5,346) (Table 1).

**Table 1:** Baseline characteristics.

	Overall	2001	2012	P trend*	P value^+^
**NVAF patients with a PPM, n**	12231	850	1344		
**Men, n (%)**	6805 (55.6)	479 (56.4)	775 (57.7)	0.003	0.030
**Median age in years (IQR)**	78 (70–84)	76 (69–83)	78 (71–84)	<0.001	0.034
**Age category, n (%)**					0.011
**<70 years**	2853 (23.3)	222 (26.1)	288 (21.4)	0.081	
**70–80 years**	4644 (38.0)	343 (40.4)	546 (40.6)	0.493	
**>80 years**	4734 (38.7)	285 (33.5)	510 (37.9)	0.028	
**Annually household income, age ≥65 years (DKK), n (%)**					<0.001
**<1 quartile (<173,650)**	2668 (25.0)	237 (33.0)	210 (17.5)	<0.001	
**1**^**st**^**– 2**^**nd**^ **quartile (173,650–230,041)**	2654 (24.9)	193 (26.8)	294 (24.5)	0.003	
**2**^**nd**^**– 3**^**rd**^ **quartile (230,041–326,604)**	2665 (25.0)	150 (20.9)	331 (27.6)	<0.001	
**≥ 3rd quartile (≥ 326,604)**	2664 (25.0)	139 (19.3)	366 (30.5)	<0.001	
**Educational level**					<0.001
**Basic & high school**	5048 (41.3)	314 (36.9)	586 (43.6)	<0.001	
**Vocational**	3311 (27.1)	157 (18.5)	452 (33.6)	<0.001	
**Higher**	1539 (12.6)	73 (8.6)	221 (16.4)	<0.001	
**AF duration, n (%)**					<0.001
**<1 year**	5615 (45.9)	472 (55.5)	570 (42.4)	<0.001	
**1–1.9 years**	1144 (9.3)	89 (10.5)	125 (9.3)	0.142	
**2–6 years**	2938 (24.0)	209 (24.6)	288 (21.4)	<0.001	
**Median AF duration in years (IQR)**	1.39 (0.07–5.09)	0.67 (0.04–3.08)	1.81 (0.12–6.58)	<0.001	<0.001
**Pacemaker type, n (%)**					
**Single chamber atrial**	505 (4.1)	73 (8.6)	5 (0.4)	<0.001	<0.001
**CRT-P**	418 (3.4)	15 (1.8)	45 (3.3)	0.048	0.044
**CRT-D**	494 (4.0)	5 (0.6)	84 (6.2)	<0.001	<0.001
**Unspecified**	1417 (11.6)	49 (5.8)	224 (16.7)	<0.001	<0.001
**Comorbidities, n (%)**					<0.001
**DM**	1704 (13.9)	61 (7.2)	226 (16.8)	<0.001	<0.001
**HF**	4104 (33.6)	312 (36.7)	394 (29.3)	0.009	0.010
**IHD**	3621 (29.6)	275 (32.4)	351 (26.1)	<0.001	<0.001
**COPD**	1318 (10.8)	65 (7.6)	151 (11.2)	<0.001	<0.001
**Ischemic stroke**	1760 (14.4)	143 (16.8)	197 (14.7)	0.188	0.116
**Bradyarrhythmia diagnosis, n (%)**					
**Sick sinus node syndrome**	5346 (43.7)	374 (44.0)	548 (40.8)	0.064	0.062
**AV-block**	3191 (26.1)	168 (19.8)	362 (26.9)	<0.001	<0.001
**Unspecified bradycardia**	2210 (18.1)	142 (16.7)	173 (12.9)	<0.001	<0.001
**Rate-lowering drugs, anti-arrhythmic drugs, and oral anticoagulants, n (%)**					
**Beta-blocker**	6169 (50.4)	324 (38.1)	779 (58.0)	<0.001	<0.001
**Digoxin**	3962 (32.4)	347 (40.8)	363 (27.0)	<0.001	<0.001
**Class 4**	1087 (8.9)	130 (15.3)	87 (6.5)	<0.001	<0.001
**Class 1C**	304 (2.5)	37 (4.4)	22 (1.6)	<0.001	<0.001
**Amiodarone**	767 (6.3)	51 (6.0)	69 (5.1)	0.009	0.011
**VKA & NOACs**	5320 (43.5)	276 (32.5)	724 (53.9)	<0.001	<0.001

Baseline characteristics for the overall cohort and year 2001 and 2012 (See S1 Table for every year and every covariate). Information on educational level and annually income is missing for some individuals. Abbreviations: IQR, interquartile range. DKK, Danish krone (national currency). AF, atrial fibrillation. AV, atrioventricular. CRT-P, Cardiac resynchronization therapy pacemaker; CRT-D, Cardiac resynchronization therapy with implantable cardiac defibrillator. COPD, chronic obstructive pulmonary disease. HF, heart failure. IHD, ischemic heart disease. DM, diabetes mellitus. Class 4, class 4 non-dihydropyridine calcium channel blocker. VKA & NOACs, vitamin-k antagonist & non-vitamin-k antagonists.

* P trend: P value test of trend in all years (2001–2012) using Cochran-Armitage trend test for categorical variables and a linear regression for continuous variables.

^**+**^ P value for statistical differences in all years (2001–2012) by Kruskal-Wallis test for continuous variables and Chi-squared test for categorical data.

### Temporal trends from 2001 to 2012

**Fig 2:**
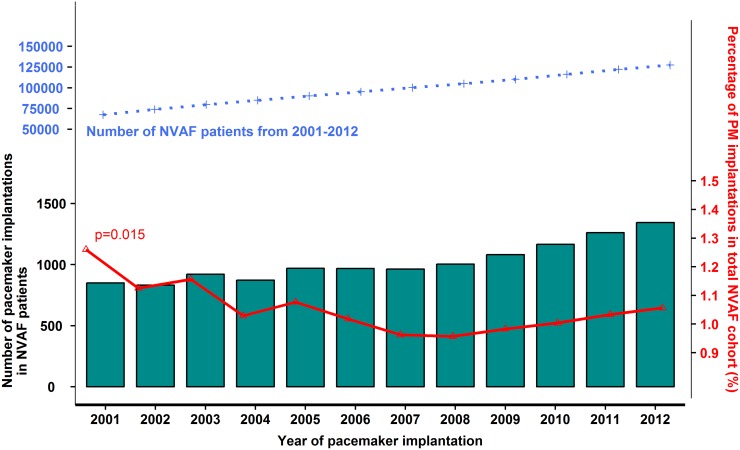
Number of NVAF patients with a PPM and number of total NVAF population. The bars show the number of NVAF patients with pacemaker implantations per year from 2001 to 2012. The blue line shows the prevalence of NVAF patients from 2001 to 2012. The red line shows the proportional percentage of NVAF patients with a PPM out of the total number of NVAF patients per year.

**Fig 3:**
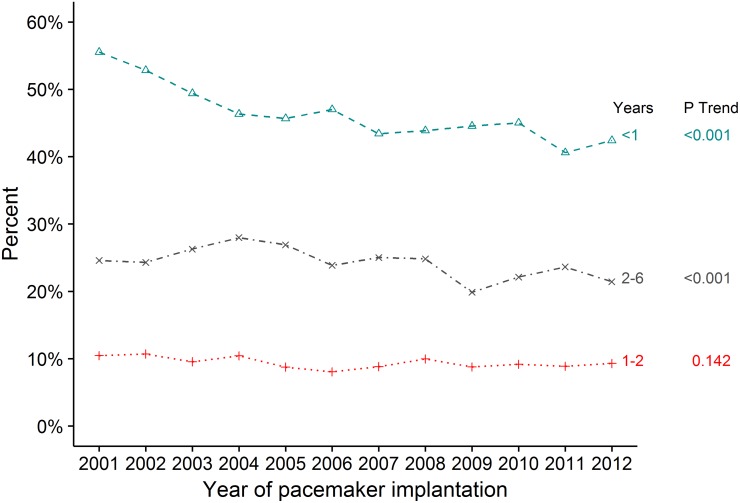
Duration of AF to pacemaker implantation. The duration of AF to pacemaker implantation shown in percent in year categories; under 1 year, 1–1.9 years, and 2–6 years of AF duration. Abbreviations: AF, atrial fibrillation.

The number of patients with NVAF and PPM implantations increased by 58.1% from 850 patients in 2001 to 1,344 patients in 2012 (p trend <0.001). In the same period, the total number of NVAF patients increased from 67,478 to 127,261 patients. Thus, the proportional amount of NVAF patients with a PPM decreased from 1.3% in 2001 to 1.1% in 2012 (p trend = 0.015) (Fig 2). From 2001 to 2012 the median (IQR) age increased from 76 (69–83) to 78 (71–84) (p trend <0.001) (Table 1). For the patients aged ≥65 years, the annual income increased; 2^nd^– 3^rd^ quartile income group increased from 20.9% to 27.6% (p trend <0.001), the ≥3^rd^ quartile income group increased from 19.3% to 30.5% (p trend <0.001). There was no significant change in income for patients under 65 years, except for the ≥3^rd^ quartile income group which also increased (S1 Table). There was a clear trend towards increased prevalence of higher educational level from 8.6% to 16.4% (p trend <0.001). Patients with AF duration less than one year to PPM implant decreased from 55.5% to 42.4% (p trend <0.001) (Fig 3). The relative use of CRT increased, while single lead pacemaker decreased (Table 1). Radiofrequency catheter ablation procedures increased from ≤0.6% to 2.5% (p trend <0.001) (S1 Table).

#### Temporal changes in comorbidities and pharmacotherapy

**Fig 4:**
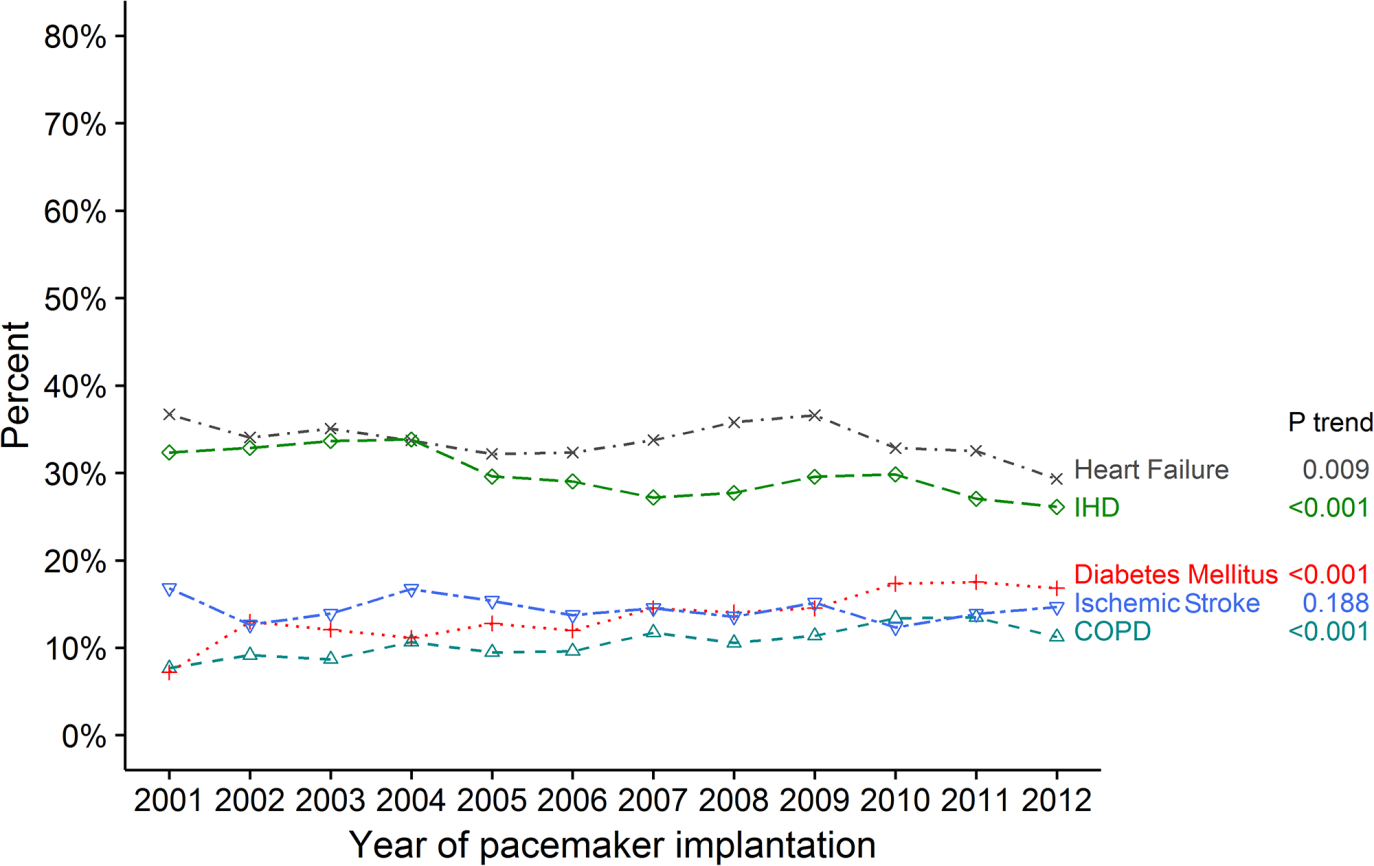
Temporal changes of prevalence in comorbidities. Temporal changes of prevalence in comorbidities in the study population from 2001 to 2012. Abbreviations: IHD, ischemic heart disease. COPD, chronic obstructive pulmonary disease.

For important comorbidities from 2001 to 2012, an increase in prevalence of DM from 7.2% to 16.8% was observed. HF decreased from 36.7% to 29.3% and IHD decreased from 32.4% to 26.1% (Fig 4). Among the diagnoses associated with implantation of PPM an increase in AV block prevalence and a decrease in unspecified bradycardia decreased was observed. No significant change in trend for of sick sinus node syndrome was observed (Table 1). Other comorbidities are shown in Table 1 and S1 Table.

**Fig 5:**
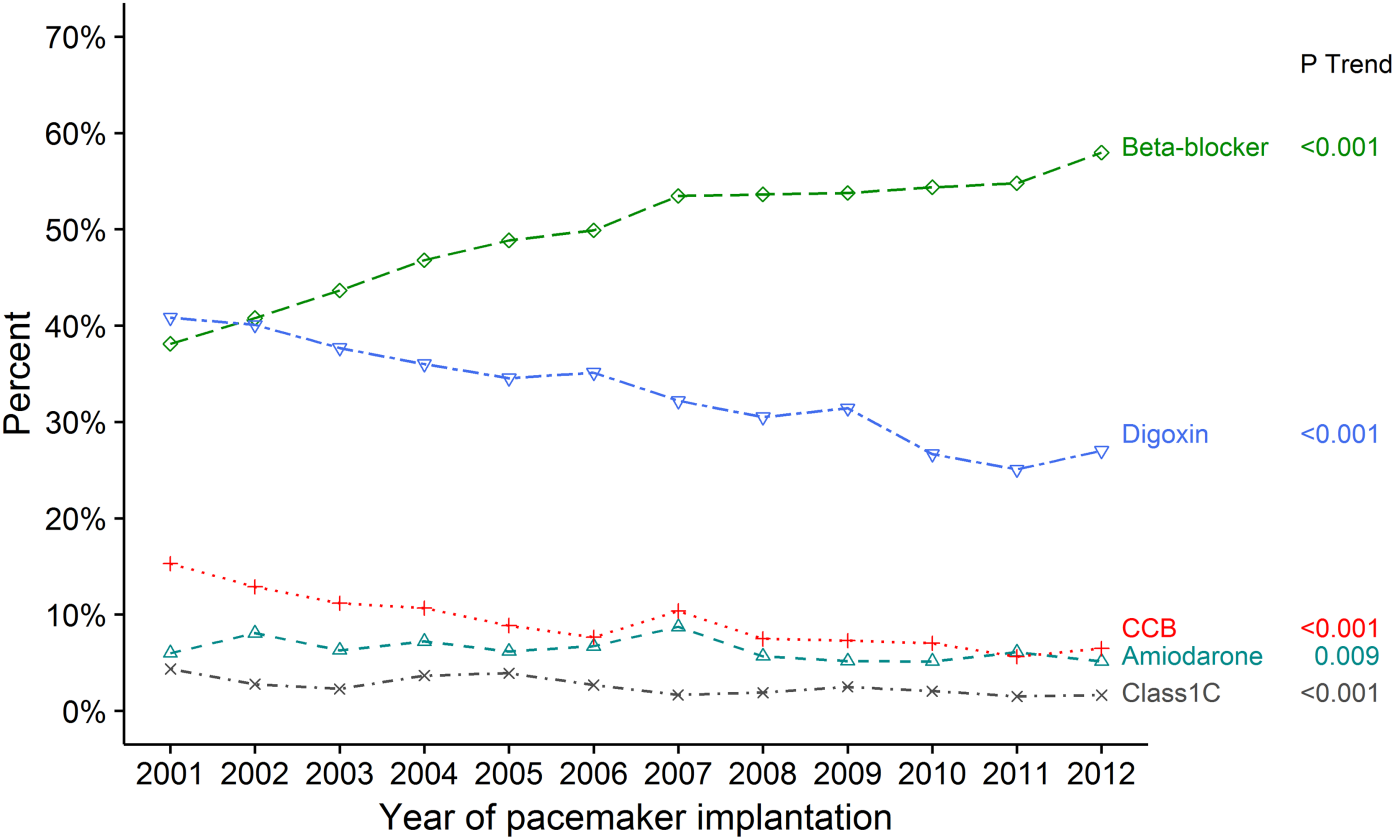
Temporal changes of prevalence in rate-lowering and anti-arrhythmic pharmacotherapy. Temporal changes of prevalence in rate-lowering and anti-arrhythmic pharmacotherapy in the study population from 2001 to 2012. CCB is class 4 non-dihydropyridine calcium channel blockers (diltiazem and verapamil). Class 1C is propafenone and flecainide.

When investigating the changes in rate-lowering and anti-arrhythmic drug use a significant 52.2% increase in beta-blocker use from 38.1% to 58.0% (p trend <0.001) was found, while the use of digoxin decreased from 40.8% to 27.0% (p trend <0.001). For amiodarone, class 4, and class 1C drugs the overall use was very low and a significant decrease in use in all three was found (Fig 5). Use of oral anticoagulants increased from 32.5% to 53.9% (p trend <0.001). Temporal trends for renin-angiotensin-system inhibitors, calcium channel blockers, loop, and non-loop diuretics are shown in S1 Table. In three subgroup analyses for patients with IHD, HF, and DM the same temporal trends for comorbidities and use of pharmacotherapies were observed.

## Discussion

This study reports temporal changes in NVAF patients with a PPM from 2001 to 2012. Our main findings were: 1) The absolute number of NVAF patients with a PPM increased, while the proportional amount of NVAF patients with a PPM decreased. 2) Almost half of the patients with a PPM, received the implantation within the first year after AF diagnosis. However, there was a trend towards increased NVAF duration before PPM over time. 3) The prevalence of DM increased while the comorbidity prevalence of IHD and HF was generally high but decreasing. 4) The use of beta-blockers increased, while the use of digoxin and anti-arrhythmic drugs decreased.

The increased number of NVAF patients with a PPM implants found in our study reflects the worldwide trend of increased use of PPM implants [17]. However, due to the increasing number of patients with NVAF the proportional amount of PPM implants decreased from 1.3% to 1.1% (p trend = 0.015). Prior studies have shown that the prevalence of AF patients with PPM ranges between 7.3%–33% which is markedly higher than the percentage found in our study. This discrepant finding could reflect differences in study cohort selection and study design; since our study used data from an unselected nationwide cohort whereas previous studies have been conducted cohort from a randomized controlled trial [1] and a cohort derived from patient records from a single hospital [18]. To our knowledge, this is the first study to have estimated the prevalence of nationwide PPMs in NVAF patients, and therefore it is not possible to determine if PPMs are underused or excessively used in the cohort.

The proportion of patients with above median quartile income and higher education level was found to be increasing during the study period. A recent U.S study investigated patients with sick sinus node syndrome and PPMs from 2003 to 2013. They found that income above median quartile had an increased risk of receiving an PPM [19], and one of their main explanations for this was patient affordability of PPM. The Danish healthcare which is available free of charge to all inhabitants independent of socioeconomic status should rule out any patient concerns about affordability. It could be hypothesized that the proportion of AF patients with PPM and high socioeconomic status increased either due to an overall increase in income and education in the AF population or due to change in which social group the physician sees fit to undergo PPM implantation. One study looking at disparities of CRT in HF patients between ethnicity and income [20] found that Afro-Americans and low-income patients were less likely to undergo CRT, indicating there could be an underuse of PPMs in low-income patients. Overall, 45.9% of the patients had PPM implantation within one year of AF diagnosis, which suggests early demasking of significant bradyarrhythmia and therefore increased risk of early PPM implantation after AF diagnosis. This might be due to early onset of anti-arrhythmic drugs side effects or comorbidities associated with sinus node remodelling such as HF [18]. There was a trend towards a longer duration of AF to PPM implantation. This could be explained by both a reduced number of conditions leading to PPM and the changes towards rate-lowering from anti-arrhythmic treatment strategy, but also by lead time bias. Lead time bias is introduced when patients get diagnosed with AF earlier in their life in 2012 than in 2001, hence the duration of AF has not increased only the time with the diagnosis. Previous radiofrequency catheter ablations were rare in this cohort, but did increase over time from under 0.6% to 2.5% which is consistent with the general trend of the AF population [21].

### Temporal changes in comorbidities

There is a known association between AF and DM; up to 20% of DM patients have AF [22]. DM increases risk of conduction pathway failure and patients with DM are more likely to get a PPM [23]. In our study, the prevalence of DM increased over time up to 16.8% in 2012. This increasing trend is expected based on increasing prevalence of DM in the Danish population [24]. A Danish study of patients with AF from 1997 to 2006 found the prevalence of DM at 9.1% [25]. Compared to the Nordic countries, a Swedish study found a total prevalence of 19.8% from 2001 to 2007 [26]. The Swedish study only included AF patients with age above 45 years, which could be why they found a higher prevalence of DM. An overall prevalence of HF of 36.8% was found, however, with a small decrease in prevalence over time. Two previous Danish studies of prevalence of HF in AF patients, have ranged between 18.8% and 23.9% [3,25] which is comparable which is comparable to the European prevalence between 22% and 42% [10]. Compared to other Nordic countries a high HF prevalence in AF was found at 46.6% in a recent study from Sweden [26]. The discrepancy can partially be explained by design; the Swedish study included both primary care and admission diagnosis. HF is associated with sinus node remodeling and has previously been identified as an independent risk factor for PPM [18]. This could to some extent explain the high prevalence of HF found in our study since all the patients in our study population already had a PPM. Our finding of temporal decreasing prevalence of HF could explain the decrease in the proportional amount of PPM implantations. The high prevalence of IHD observed in our study is in accordance with a German study, the AFFIRM trial, the Swedish study and the overall reported prevalence of IHD in AF ranging from 17%-46.5% [1,26–28]. Among bradycardia diagnoses indicating the need of PPM, sick sinus node syndrome was the highest with a prevalence of 43.7%. Sick sinus node syndrome has previously been reported at 79% of bradycardia patients requiring pacemakers [29] which supports that sick sinus node syndrome is the most prevalent indication of PPM in AF patients.

### Temporal changes in pharmacotherapy

The use of pharmacotherapy in NVAF patients with a PPM has changed markedly over time. Beta-blocker use increased by 52.2%, while use of digoxin, amiodarone, class 1C, and class 4 anti-arrhythmic drugs decreased. Similarly, an increased use of beta-blockers and declining use of digoxin and anti-arrhythmic drugs has been reported in earlier in studies among Danish patients with AF from 1995 to 2004 [3] and from 2000 to 2009 [21]. The same trend in pharmacotherapy was also found in Swedish and U.S patients with AF [30,31] during the same period of time, suggesting a global shift in treatment strategy. The first ACC/AHA/ESC guidelines for AF treatment was published in 2001. In these guidelines, no specific strategy in the choice between rate-lowering or anti-arrhythmic drugs was recommended, thus these guidelines cannot explain the early shift towards increased use of beta-blockers found in this study. The increased use of beta-blockers could be due to a “carry-over effect”, where beta-blockers have shown benefits in patients with HF or IHD and the treatment is continued after AF diagnosis [3,32]. Besides the expanding indication for beta-blocker treatment, trials have favored the treatment of AF towards the rate-lowering treatment strategy. The AFFIRM trial in 2002 concluded no benefit of rhythm control over rate control and as rate control is associated with less side-effects this could explain the shift towards an increased use of beta-blockers and decrease in amiodarone [1]. Later the RACE trial in 2010 concluded that strict rate control was not superior to lenient rate control. The trial may have led to reduced dosage of rate-lowering drugs, and thus decreasing the amount of iatrogenic brady-arrhythmias necessitating a PPM [2]. In an observational study of ischemic patients with AF the risk of PPM increased with both amiodarone (adjusted OR 2.14, 95% CI: 1.30–3.54) and digoxin use (adjusted OR 1.78, 95% CI 1.37–2.31) [5]. Since both amiodarone and digoxin use decreased over time in our study this could to some extend explain the decrease in the proportional amount of NVAF patients with a PPM.

### Strengths and limitations

The registries used for this study comprise an unselected population of patients and are not affected by selection bias to certain hospital centres, health insurance systems or age groups. Thus, this study data reflects real clinical practice on a nationwide scale. Despite these strengths, there were some limitations: the non-availability of a precise indication for PPM implantation, and thus no data on the underuse or excessively use of PPM implantation in the NVAF cohort. Other limitations included frequency of AF episodes and AF type (paroxysmal, persistent or permanent), indication for pharmacotherapy, and adverse reactions. Absence of data on HbA1c might have excluded undiagnosed DM.

## Conclusion

From 2001 to 2012, the absolute number NVAF patients with a PPM increased while the proportional amount decreased. The number of patients who received a PPM within one year of AF diagnosis decreased. The prevalence of DM increased, while the prevalence of IHD and HF was high but decreasing. The use of beta-blockers increased markedly, while use of digoxin and anti-arrhythmic drugs decreased over time. These findings provide key knowledge of NVAF patients with a PPM.

## Supporting information

S1 Table Baseline characteristics for all covariates from 2001 to 2012. **+** P value for statistical differences in all years (2001–2012) by Kruskal-Wallis test for continuous variables and Chi-squared test for categorical data. Information on educational level and annually income is missing for some individuals Abbreviations: IQR, interquartile range. DKK, Danish krone (national currency). COPD, chronic obstructive pulmonary disease. HF, heart failure, IHD, ischemic heart disease. DM, diabetes mellitus. Class 4, class 4 non-dihydropyridine calcium channel blocker. RAS inhibitor and ARBs, renin-angiotensin system inhibitor and angiotensin II receptor blockers. DC, electrical cardioversion. PCI, percutaneous coronary intervention. CABG, coronary arterial bypass graft. AF, atrial fibrillation. AV, atrio-ventricular. CRT-P, Cardiac resynchronization therapy pacemaker. CRT-D, Cardiac resynchronization therapy with implantable cardiac defibrillator.
(DOCX)

S2 Table ICD-10, ICD-8, NCSP, and ATC codes. Discharge codes by ICD-10 or ICD-8. Procedure codes from NCSP and pharmacotherapy use by ATC codes. Abbreviations: CRT-P, Cardiac resynchronization therapy pacemaker. CRT-D, Cardiac resynchronization therapy with implantable cardiac defibrillator.
(DOCX)
