# Supplementary material for: Patients with atrial fibrillation and permanent pacemaker: Temporal changes in patient characteristics and pharmacotherapy
Source: PLoS One. 2018 Mar 28;13(3):e0195175. doi: 10.1371/journal.pone.0195175 (PMC5874078; doi:10.1371/journal.pone.0195175)
Supplement: S1 Table — + P value for statistical differences in all years (2001–2012) by Kruskal-Wallis test for continuous variables and Chi-squared test for categorical data. Information on educational level and annually income is missing for some individuals Abbreviations: IQR, interquartile range. DKK, Danish krone (national currency). COPD, chronic obstructive pulmonary disease. HF, heart failure, IHD, ischemic heart disease. DM, diabetes mellitus. Class 4, class 4 non-dihydropyridine calcium channel blocker. RAS inhibitor and ARBs, renin-angiotensin system inhibitor and angiotensin II receptor blockers. DC, electrical cardioversion. PCI, percutaneous coronary intervention. CABG, coronary arterial bypass graft. AF, atrial fibrillation. AV, atrio-ventricular. CRT-P, Cardiac resynchronization therapy pacemaker. CRT-D, Cardiac resynchronization therapy with implantable cardiac defibrillator. (DOCX) [file pone.0195175.s001.docx]

|  | **Overall** | **2001** | **2002** | **2003** | **2004** | **2005** | **2006** | **2007** | **2008** | **2009** | **2010** | **2011** | **2012** | **P value^+^** |
| --- | --- | --- | --- | --- | --- | --- | --- | --- | --- | --- | --- | --- | --- | --- |
| Number of patients | 12231 | 850 | 831 | 921 | 872 | 970 | 968 | 963 | 1003 | 1082 | 1166 | 1261 | 1344 |  |
| male, n (%) | 6805 (55.6) | 479 (56.4) | 428 (51.5) | 503 (54.6) | 464 (53.2) | 548 (56.5) | 530 (54.8) | 522 (54.2) | 553 (55.1) | 587 (54.3) | 667 (57.2) | 749 (59.4) | 775 (57.7) | 0.030 |
| Median age in years(IQR) | 78 (70- 84) | 76 (69- 83) | 78 (70- 83) | 78 (70- 84) | 78 (71- 84) | 78 (70- 84) | 78 (70- 84) | 79 (70- 84) | 78 (70- 84) | 78 (70- 84) | 78 (71- 84) | 77 (70- 84) | 78 (71- 84) | 0.034 |
| **Annually household income <65 age (DKK), n (%)** | | | | | | | | | | | | | | 0.014 |
| <1 quartile (<308,520) | 394 (25.0) | 35 (26.7) | 33 (30.0) | 30 (23.1) | 33 (28.0) | 27 (19.7) | 48 (32.9) | 42 (32.8) | 31 (24.6) | 25 (19.2) | 22 (18.6) | 33 (21.0) | 35 (24.5) |  |
| 1^st^ – 2^nd^ quartile (308,520-478,374) | 393 (25.0) | 40 (30.5) | 22 (20.0) | 32 (24.6) | 36 (30.5) | 31 (22.6) | 33 (22.6) | 22 (17.2) | 37 (29.4) | 37 (28.5) | 29 (24.6) | 40 (25.5) | 34 (23.8) |  |
| 2^nd^ – 3^rd^ quartile (478,374-643,428) | 393 (25.0) | 34 (26.0) | 29 (26.4) | 44 (33.8) | 31 (26.3) | 42 (30.7) | 26 (17.8) | 27 (21.1) | 30 (23.8) | 26 (20.0) | 32 (27.1) | 39 (24.8) | 33 (23.1) |  |
| ≥ 3rd quartile (≥ 643,428) | 394 (25.0) | 22 (16.8) | 26 (23.6) | 24 (18.5) | 18 (15.3) | 37 (27.0) | 39 (26.7) | 37 (28.9) | 28 (22.2) | 42 (32.3) | 35 (29.7) | 45 (28.7) | 41 (28.7) |  |
| **Annually household income, ≥65 age (DKK), n (%)** | | | | | | | | | | | | | | <0.001 |
| <1 quartile (<173,650) | 2668 (25.0) | 237 (33.0) | 237 (32.9) | 237 (30.0) | 232 (30.8) | 229 (27.5) | 228 (27.8) | 219 (26.2) | 201 (22.9) | 221 (23.2) | 213 (20.3) | 204 (18.5) | 210 (17.5) |  |
| 1^st^ – 2^nd^ quartile (173,650-230,041) | 2654 (24.9) | 193 (26.8) | 196 (27.2) | 226 (28.6) | 188 (25.0) | 212 (25.5) | 185 (22.6) | 215 (25.7) | 208 (23.7) | 245 (25.8) | 250 (23.9) | 242 (21.9) | 294 (24.5) |  |
| 2^nd^ – 3^rd^ quartile (230,041-326,604) | 2665 (25.0) | 150 (20.9) | 142 (19.7) | 161 (20.4) | 170 (22.6) | 192 (23.1) | 213 (26.0) | 200 (24.0) | 237 (27.1) | 240 (25.2) | 273 (26.0) | 356 (32.2) | 331 (27.6) |  |
| ≥ 3rd quartile (≥ 326,604) | 2664 (25.0) | 139 (19.3) | 146 (20.2) | 167 (21.1) | 163 (21.6) | 199 (23.9) | 194 (23.7) | 201 (24.1) | 230 (26.3) | 245 (25.8) | 312 (29.8) | 302 (27.4) | 366 (30.5) |  |
| **Educational level, n (%)** | | | | | | | | | | | | | | <0.001 |
| Basic or high school | 5048 (41.3) | 314 (36.9) | 302 (36.3) | 319 (34.6) | 360 (41.3) | 362 (37.3) | 379 (39.2) | 428 (44.4) | 432 (43.1) | 491 (45.4) | 515 (44.2) | 560 (44.4) | 586 (43.6) |  |
| Vocational | 3311 (27.1) | 157 (18.5) | 159 (19.1) | 210 (22.8) | 185 (21.2) | 251 (25.9) | 273 (28.2) | 247 (25.6) | 292 (29.1) | 295 (27.3) | 370 (31.7) | 420 (33.3) | 452 (33.6) |  |
| Higher | 1539 (12.6) | 73 (8.6) | 86 (10.3) | 102 (11.1) | 73 (8.4) | 119 (12.3) | 117 (12.1) | 117 (12.1) | 138 (13.8) | 144 (13.3) | 164 (14.1) | 185 (14.7) | 221 (16.4) |  |
|  |  |  |  |  |  |  |  |  |  |  |  |  |  |  |
| **Comorbidities, n (%)** | | | | | | | | | | | | | | |
| Ischemic stroke | 1760 (14.4) | 143 (16.8) | 105 (12.6) | 128 (13.9) | 146 (16.7) | 149 (15.4) | 133 (13.7) | 140 (14.5) | 136 (13.6) | 164 (15.2) | 144 (12.3) | 175 (13.9) | 197 (14.7) | 0.116 |
| COPD | 1318 (10.8) | 65 (7.6) | 76 (9.1) | 80 (8.7) | 93 (10.7) | 92 (9.5) | 93 (9.6) | 113 (11.7) | 106 (10.6) | 123 (11.4) | 156 (13.4) | 170 (13.5) | 151 (11.2) | <0.001 |
| Heart Failure | 4104 (33.6) | 312 (36.7) | 283 (34.1) | 323 (35.1) | 294 (33.7) | 312 (32.2) | 313 (32.3) | 325 (33.7) | 359 (35.8) | 397 (36.6) | 383 (32.8) | 410 (32.5) | 394 (29.3) | 0.010 |
| IHD | 3621 (29.6) | 275 (32.4) | 273 (32.9) | 310 (35.1) | 295 (33.8) | 287 (29,6) | 281 (29.0) | 262 (27.2) | 278 (27.7) | 320 (29.6) | 348 (29.8) | 341 (27.0) | 351 (26.1) | <0.001 |
| Diabetes mellitus | 1704 (13.9) | 61 (7.2) | 108 (13.0) | 111 (12.1) | 97 (11.1) | 124 (12.8) | 116 (12.0) | 140 (14.5) | 141 (14.1) | 157 (14.5) | 202 (17.3) | 221 (17.5) | 226 (16.8) | <0.001 |
| **Anti-arrhythmic drugs, n (%)** | | | | | | | | | | | | | | |
| Digoxin | 3962 (32.4) | 347 (40.8) | 333 (40.1) | 347 (37.7) | 314 (36.0) | 335 (34.5) | 340 (35.1) | 310 (32.2) | 306 (30.5) | 340 (31.4) | 311 (26.7) | 316 (25.1) | 363 (27.0) | <0.001 |
| Class1C | 304 (2.5) | 37 (4.4) | 23 (2.8) | 21 (2.3) | 32 (3.7) | 38 (3.9) | 26 (2.7) | 16 (1.7) | 19 (1.9) | 27 (2.5) | 24 (2.1) | 19 (1.5) | 22 (1.6) | <0.001 |
| Amiodarone | 767 (6.3) | 51 (6.0) | 67 (8.1) | 58 (6.3) | 63 (7.2) | 60 (6.2) | 65 (6.7) | 84 (8.7) | 57 (5.7) | 56 (5.2) | 60 (5.1) | 77 (6.1) | 69 (5.1) | 0.011 |
| Beta blocker | 6169 (50.4) | 324 (38.1) | 339 (40.8) | 402 (43.6) | 408 (46.8) | 474 (48.9) | 483 (49.9) | 515 (53.5) | 538 (53.6) | 582 (53.8) | 634 (54.4) | 691 (54.8) | 779 (58.0) | <0.001 |
| Class IV | 1087 (8.9) | 130 (15.3) | 107 (12.9) | 103 (11.2) | 93 (10.7) | 86 ( 8.9) | 74 (7.6) | 100 (10.4) | 75 (7.5) | 79 (7.3) | 82 (7.0) | 71 (5.6) | 87 (6.5) | <0.001 |
| **Other cardiovascular drugs, n (%)** | | | | | | | | | | | | | | |
| Loop diuretics | 5181 (42.3) | 343 (40.4) | 365 (43.9) | 383 (41.6) | 402 (46.1) | 404 (41.6) | 401 (41.4) | 457 (47.5) | 422 (42.1) | 460 (42.5) | 469 (40.2) | 521 (41.3) | 554 (41.2) | 0.026 |
| Non-loop diuretics | 5023 (41.1) | 268 (31.5) | 326 (39.2) | 359 (39.0) | 369 (42.3) | 414 (42.7) | 374 (38.6) | 419 (43.5) | 450 (44.9) | 471 (43.5) | 498 (42.7) | 518 (41.1) | 557 (41.4) | <0.001 |
| RAS inhibitors | 6061 (49.6) | 290 (34.1) | 315 (37.9) | 390 (42.3) | 383 (43.9) | 439 (45.3) | 467 (48.2) | 509 (52.9) | 537 (53.5) | 591 (54.6) | 671 (57.5) | 714 (56.6) | 755 (56.2) | <0.001 |
| Antiadrenerge | 260 (2.1) | 15 (1.8) | 12 (1.4) | 18 (1.9) | 21 (2.4) | 18 (1.9) | 16 (1.7) | 17 (1.8) | 21 (2.1) | 33 (3.0) | 29 (2.5) | 32 (2.5) | 28 (2.1) | 0.439 |
| CCBs (%) | 3524 (28.8) | 244 (28.7) | 229 (27.6) | 248 (26.9) | 250 (28.7) | 261 (26.9) | 239 (24.7) | 290 (30.1) | 288 (28.7) | 324 (29.9) | 379 (32.5) | 354 (28.1) | 418 (31.1) | 0.009 |
| Oral anticoagulants | 5320 (43.5) | 276 (32.5) | 243 (29.5) | 329 (35.7) | 353 (40.5) | 398 (41.0) | 440 (45.5) | 463 (48.1) | 463 (46.2) | 477 (44.1) | 532 (45.6) | 620 (49.2) | 724 (53.9) | <0.001 |
| **Procedures, n (%)** | | | | | | | | | | | | | | |
| DC | 1224 (10.0) | 84 (9.9) | 71 (8.5) | 100 (10.9) | 71 (8.1) | 95 (9.8) | 97 (10.0) | 99 (10.3) | 84 (8.4) | 104 (9.6) | 116 (9.9) | 148 (11.7) | 155 (11.5) | 0.106 |
| PCI | 801 (6.6) | 36 (4.2) | 31 (3.7) | 53 (5.8) | 48 (5.5) | 66 (6.8) | 77 (8.0) | 60 (6.2) | 76 (7.6) | 80 (7.4) | 87 (7.5) | 91 (7.2) | 96 (7.1) | 0.001 |
| CABG | 366 (3.0) | 34 (4.0) | 25 (3.0) | 50 (5.4) | 18 (2.1) | 30 (3.1) | 24 (2.5) | 23 (2.4) | 24 (2.4) | 43 (4.0) | 23 (2.0) | 33 (2.6) | 39 (2.9) | <0.001 |
| Ablation | 237 (1.9) | ≤ 3 | 5 (0.6) | ≤ 3 | 8 (0.9) | 17 (1.8) | 19 (2.0) | 23 (2.4) | 24 (2.4) | 27 (2.5) | 35 (3.0) | 39 (3.1) | 34 (2.5) | <0.001 |
| **Atrial fibrillation duration in year categories, n (%)** | | | | | | | | | | | | | | <0.001 |
| <1 year | 5615 (45.9) | 472 (55.5) | 439 (52.8) | 455 (49.4) | 404 (46.3) | 443 (45.7) | 455 (47.0) | 418 (43.4) | 440 (43.9) | 482 (44.5) | 525 (45.0) | 512 (40.6) | 570 (42.4) |  |
| 1-1.9 years | 1144 (9.3) | 89 (10.5) | 89 (10.5) | 88 (9.6) | 91 (10.4) | 85 (8.8) | 78 (8.1) | 85 (8.8) | 100 (10.0) | 95 (8.8) | 107 (9.2) | 112 (8.9) | 125 (9.3) |  |
| 2-6 years | 2938 (24.0) | 209 (24.6) | 202 (24.3) | 242 (26.3) | 244 (28.0) | 261 (26.9) | 231 (23.9) | 241 (25.0) | 249 (24.8) | 215 (19.9) | 258 (22.1) | 298 (23.6) | 288 (21.4) |  |
| AF duration (median [IQR]) | 1.39 (0.07-5.09) | 0.67 (0.04-3.08) | 0.79 (0.04-3.51) | 1.05 (0.06-4.11) | 1.31 (0.05-4.42) | 1.41 (0.07-4.71) | 1.30 (0.05-5.35) | 1.68 (0.10-5.51) | 1.51 (0.10-5.22) | 1.61 (0.11-6.34) | 1.54 (0.08-5.57) | 2.06 (0.16-6.57) | 1.81 (0.12-6.58) | <0.001 |
| **Bradyarrhythmia diagnosis, n (%)** | | | | | | | | | | | | | | |
| Sick sinus syndrome | 5346 (43.7) | 374 (44.0) | 385 (46.3) | 421 (45.7) | 370 (42.4) | 412 (42.5) | 421 (43.5) | 430 (44.7) | 433 (43.2) | 512 (47.3) | 518 (44.4) | 522 (41.4) | 548 (40.8) | 0.062 |
| AV-block | 3191 (26.1) | 168 (19.8) | 197 (23.7) | 222 (24.1) | 220 (25.2) | 216 (22.3) | 274 (28.3) | 300 (31.2) | 285 (28.4) | 315 (29.1) | 318 (27.3) | 314 (24.9) | 362 (26.9) | <0.001 |
| Unspecified bradycardia | 2210 (18.1) | 142 (16.7) | 168 (20.2) | 220 (23.9) | 204 (23.4) | 218 (22.5) | 186 (19.2) | 185 (19.2) | 171 (17.0) | 173 (16.0) | 201 (17.2) | 169 (13.4) | 173 (12.9) | <0.001 |
| **Pacemaker type, n (%)** | | | | | | | | | | | | | | |
| Single chamber  atrial | 505 (4.1) | 73 (8.6) | 59 (7.1) | 70 (7.6) | 72 (8.3) | 61 (6.3) | 53 (5.5) | 31 (3.2) | 24 (2.4) | 20 (1.8) | 26 (2.2) | 11 (0.9) | 5 (0.4) | <0.001 |
| Single chamber ventricle | 4574 (37.4) | 339 (39.9) | 331 (39.8) | 414 (45.0) | 393 (45.1) | 404 (41.6) | 373 (38.5) | 362 (37.6) | 385 (38.4) | 372 (34.4) | 401 (34.4) | 379 (30.1) | 421 (31.3) | <0.001 |
| Dual chamber | 4823 (39.4) | 369 (43.4) | 373 (44.9) | 356 (38.7) | 309 (35.4) | 304 (31.3) | 331 (34.2) | 356 (37.0) | 361 (36.0) | 438 (40.5) | 523 (44.9) | 538 (42.7) | 565 (42.0) | <0.001 |
| CRT-P | 418 (3.4) | 15 (1.8) | 29 (3.5) | 30 (3.3) | 36 (4.1) | 34 (3.5) | 32 (3.3) | 28 (2.5) | 28 (2.8) | 39 (3.6) | 39 (3.3) | 63 (5.0) | 45 (3.3) | 0.044 |
| CRT-D | 494 (4.0) | 5 (0.6) | 10 (1.2) | 13 (1.4) | 14 (1.6) | 29 (3.0) | 24 (2.5) | 42 (4.4) | 52 (5.2) | 66 (6.1) | 57 (4.9) | 98 (7.8) | 84 (6.2) | <0.001 |
| Unspecified | 1417 (11.6) | 49 (5.8) | 29 (3.5) | 38 (4.1) | 48 (5.5) | 138 (14.2) | 155 (16.0) | 144 (15.0) | 153 (15.3) | 147 (13.6) | 120 (10.3) | 172 (13.6) | 224 (16.7) | <0.001 |
