# Supplementary material for: Patients with atrial fibrillation and permanent pacemaker: Temporal changes in patient characteristics and pharmacotherapy
Source: PLoS One. 2018 Mar 28;13(3):e0195175. doi: 10.1371/journal.pone.0195175 (PMC5874078; doi:10.1371/journal.pone.0195175)
Supplement: S2 Table — Discharge codes by ICD-10 or ICD-8. Procedure codes from NCSP and pharmacotherapy use by ATC codes. Abbreviations: CRT-P, Cardiac resynchronization therapy pacemaker. CRT-D, Cardiac resynchronization therapy with implantable cardiac defibrillator. (DOCX) [file pone.0195175.s002.docx]

| **Covariate** | **ICD-10, ICD-8, NCSP, and ATC codes** |
| --- | --- |
| **Atrial fibrillation** | ICD-10: I48  ICD-8: 42794, 42795 |
| **Non-valvular atrial fibrillation** | Presence of: I48, 42794, 42795  With absence of: Rheumatic heart valve disease:  ICD8: 4240, 4241, 39500-39502, 39508, 39509, 39600-39604, 39608, 39609  ICD10: Z952, Z954, I05, I06, I080A, I081A, I082A, I083A Prostatic heart valve  KFKD, KFKH, KFMD, KFMH, KFGE, KFJF |
| **Ischemic stroke** | Ischemic: I63, I64  transitional cerebral ischemia: G459, G458  Systemic embolism and thrombosis: I74 |
| **Heart failure** | Cardiomyopathy: I42  Heart failure: I50, I110  Lung edema: J81, |
| **Ischemic heart disease** | Ischemic heart disease: I20-I25   - Angina pectoris: I20 - Acute myocardial infarction: I21, I22 - Complications to acute myocardial infarction: I23, - Other forms of ischemic heart disease: I24, I25 |
| **Chronic obstructive pulmonary disease** | J42, J43, J44 |
| **Diabetes mellitus** | Insulin: A10A  Non-Insulin: A10B |
| **Sick sinus node syndrome** | I495, tachycardia-bradycardia syndrome: I495b |
| **Atrio-ventricular block** | 1^st^ degree: I440, 2^nd^ degree type 1: I441b, 2^nd^ degree type 2: I441C, 3^rd^ degree: I442, unspecified: I443 |
| **Unspecified bradycardia** | DR001 |
| **Permanent pacemaker implantation** | procedure code; KFPE00, KFPE10, KFPE20, KFPE96; surgery code: BFCA0-08, BFCA6-63, BFCB03  Atrial single chamber: BFCA02, BFCA62, BFCA64, KFPE10  Ventricle single chamber: BFCA01, BFCA08, BFCA61, BFCA63, KFPE00  Dual chamber: BFCA03, BFCA07, KFPE20  CRT-P: BFCA04, BFCA05, BFCA06  CRT-D: BFCB03  Unspecified: KFPE96, BFCA0, BFCA6 |
| **Cardioversion** | Atrial fibrillation: BFFA00, BFFA01, BFFA04 |
| **Ablation** | Atrial fibrillation: BFFB04 |
| **Anti-arrhythmic drugs** | Amiodarone: C01BD01  Class 1C: C01BC |
| **Rate-lowering drugs** | Digoxin: C01AA  Beta-blockers: C07A, C07B, C07C, C07D, C07F  Class 4 calcium channel blockers: C08D |
| **Other cardiovascular medicine** | Non-Loop diuretics:  Thiazides C02L, C02DA, C07B, C07D,  C09XA52, C03A, C03EA;  Low-ceiling diuretics (excl. thiazides): C03B, C03X, C07C, C08G, C09BA, C09DA, Potassium-sparing agents (spiron): C03D, C03E, C03EB  Loop: High-ceiling diuretics C03C, C03EB  Antiadrenergic agents: C02A, C02B, C02C  Vasodilators: C02DB, C02DD, C02DG  Calcium channel blockers: C08, C09BB, C09DB  Renin angiotensin system inhibitors & Angiotensin II receptor blockers: C09AA, C09BA, C09BB, C09CA, C09DA, C09DB, C09XA02, C09XA52  Oral anticoagulants: Vitamin-k antagonist; B01AA, Non-vitamin K-antagonist: B01AE, B01AF |
